# A study on the antibacterial efficacy and action mechanism of clove against *Helicobacter pylori*

**DOI:** 10.3389/fphar.2026.1802776

**Published:** 2026-05-11

**Authors:** Xuanru Kang, Yangchenze Fan, Xianmei Meng, Zhenyu Jiang, Chi Wang, Jiaying Zhu, Yuanyuan Nian

**Affiliations:** 1 Baotou Medical College, Inner Mongolia University of Science and Technology, Baotou, China; 2 The Second Affiliated Hospital of Baotou Medical College, Inner Mongolia University of Science and Technology, Baotou, China

**Keywords:** antibacterial activity, clove, *H. pylori*, network pharmacology, toll-like receptor signaling pathway

## Abstract

**Background:**

This study aimed to investigate the anti-*Helicobacter pylori* (*H. pylori*) effects of clove (*Syzygium aromaticum*) and its underlying mechanisms.

**Method:**

Network pharmacology was used to identify potential targets of clove against *H. pylori*, followed by GO and KEGG enrichment analyses. Molecular docking was performed to evaluate the binding of key clove-derived compounds to core targets. *In vitro* antimicrobial susceptibility testing against the *H. pylori* SS1 strain was conducted to determine antibacterial activity, and an *H. pylori* infected rat model was used to assess the *in vivo* anti-inflammatory effects of clove.

**Result:**

Network pharmacology identified six bioactive components and 19 overlapping targets, among which TP53, MMP9, IL1B, IL10, and TNF were central nodes. Docking analysis showed favorable binding between major compounds and core targets, with quercetin exhibiting the lowest binding energy toward MMP9 (−10.5). *In vitro*, kaempferol, quercetin, and stigmasterol inhibited *H. pylori* SS1, with MIC/MBC values of 0.5/1, 2.5/10, and 3.13/12.52 mg/mL, respectively, whereas strictosamide showed weak activity (MIC 10 mg/mL, no detectable MBC) and β-sitosterol did not exhibit quantifiable MIC or MBC values within the tested range. *In vivo*, clove treatment dose-dependently reduced gastric TNF-α, IL-12, IL-17, IL-23, and IFN-γ levels in *H. pylori* infected rats and was associated with increased IL-10 expression, particularly in the medium- and high-dose groups at both the protein and mRNA levels. Clove also reduced the elevated protein and mRNA expression levels of IL-1β, MMP9, TP53, and TLR4 in gastric tissue.

**Conclusion:**

These findings suggest that clove contains constituents with direct *in vitro* anti-*H. pylori* activity and may exert anti-inflammatory and gastroprotective effects in *H. pylori* infected rats. The observed changes in cytokines and inflammation-related molecules support the potential involvement of TNF- and TLR4-associated signaling, although further studies are required to verify direct pathway regulation and *in vivo* antibacterial mechanisms.

## 1 Introduction

*Helicobacter pylori* (*H. pylori*) infection poses a significant threat to human digestive health (Malfertheiner et al., 2023). Currently, bismuth-based quadruple therapy remains one of the main first-line strategies for *H. pylori* eradication in clinical practice (Chey et al., 2024). However, with the widespread and often inappropriate use of antibiotics, antimicrobial resistance in *H. pylori* has become increasingly prominent (Hasanuzzaman et al., 2024). Therefore, the identification of safe and effective treatment strategies that are less likely to induce drug resistance is of considerable clinical significance. As an integral part of traditional medicine, Chinese and Mongolian medicines have shown broad potential in treating infectious diseases due to their unique advantages of “multi-component, multi-target, and holistic regulation” (Zheng et al., 2022). Numerous studies indicate that single or compound formulations of these medicines can not only directly inhibit the growth of *H. pylori*, but also exert synergistic therapeutic effects through multiple pathways, such as modulating the inflammatory microenvironment, restoration of the gastric mucosal barrier, and improvement of clinical symptoms (Zhang P. et al., 2025; Shen et al., 2021). These treatments may help reduce treatment-related adverse effects and offer complementary value in the comprehensive management of *H. pylori* infection, particularly in the context of increasing antibiotic resistance (Bao et al., 2022).

Our research team has previously conducted preliminary work in this field. Antimicrobial susceptibility testing of 13 commonly used Chinese and Mongolian medicinal herbs demonstrated that clove (*Syzygium aromaticum*) exhibited stable inhibitory activity against both the *H. pylori* reference strain (ATCC 43504) and clinical isolates. The inhibition zones were 11.13 mm for the reference strain and 11.08 mm for the clinical isolates, both meeting the criteria against intermediate susceptibility (Liu et al., 2024). Previous phytochemical studies have shown that clove contains multiple bioactive constituents, including quercetin and kaempferol, some of which have been reported to possess antimicrobial or related pharmacological activities (Cortés-Rojas et al., 2014; Jan et al., 2022). These findings suggest that clove may be a promising candidate for anti-*H. pylori* research. However, the specific material basis, core active constituents, and molecular mechanisms underlying its anti-*H. pylori* effects remain insufficiently understood.

To address these questions, the present study established a comprehensive research framework integrating network pharmacology, molecular docking, *in vitro* antimicrobial susceptibility testing, and *in vivo* pharmacodynamic evaluation. The aim was to decipher the coordinated regulatory network of “components, targets, and pathways” underlying the anti-*H. pylori* effects of clove, to clarify its synergistic antibacterial and anti-inflammatory mechanisms, and to provide experimental evidence for the development of clove-based Chinese and Mongolian medicinal formulations against *H. pylori*. Furthermore, this study aimed to provide novel insights and potential strategies for the treatment of antibiotic-resistant *H. pylori* strains.

## 2 Materials and methods

### 2.1 Materials

#### 2.1.1 Strains

*Helicobacter pylori* SS1 was purchased from Hangzhou Hong-Sai Biotechnology Co., Ltd. and stored at −80 °C until use.

#### 2.1.2 Animal model

A total of 50 healthy specific pathogen-free male SD rats were obtained from SPF (Beijing) Biotechnology Co., Ltd (license number: SCXK [Beijing] 2024-0001). All experimental procedures were reviewed and approved by the Ethics Committee of The Second Affiliated Hospital of Baotou Medical College, Inner Mongolia University of Science and Technology (approval number: 2024-ZX-103).

#### 2.1.3 Drugs and reagents

Clove was provided by the Department of Traditional Chinese Medicine Pharmacy, The Second Affiliated Hospital of Baotou Medical College, Inner Mongolia University of Science and Technology. Strictosamide_qt, quercetin, β-sitosterol, kaempferol, and stigmasterol were purchased from Beijing InnoChem Science & Technology Co., Ltd. Enzyme-linked immunosorbent assay (ELISA) kits for IL-12, IL-17, IL-23, IFN-γ, and TNF-α were obtained from Wuhan Fine Biotech Co., Ltd. Antibodies against GAPDH, IL-10, MMP9, TP53, and TLR4 were purchased from Wuhan Sanying Biotechnology Co., Ltd., and an antibody against IL-1β was obtained from Abcam.

### 2.2 Network pharmacology

#### 2.2.1 Identification of clove targets against *Helicobacter pylori*

The chemical constituents of clove were retrieved from the Traditional Chinese Medicine Systems Pharmacology Database and Analysis Platform (TCMSP; https://www.tcmsp-e.com/). Potential bioactive components were screened according to the criteria of oral bioavailability (OB) ≥ 30% and drug-likeness (DL) ≥ 0.18, based on previously published studies (Yue et al., 2023). The 2D structures of the selected compounds were downloaded as SDF files from the PubChem database (https://pubchem.ncbi.nlm.nih.gov/) and then uploaded to the SwissTargetPrediction database (http://www.swisstargetprediction.ch/), PharmMapper database (http://www.lilab-ecust.cn/pharmmapper/), and SuperPred database (https://prediction.charite.de/) for target prediction, with the species restricted to *Homo sapiens*. In SwissTargetPrediction, only targets with positive probability were retained, whereas all predicted human-related targets from PharmMapper and SuperPred were included. After standardization using the UniProt database (https://www.uniprot.org/), duplicate targets were removed to obtain the putative targets of clove. Genes associated with *H. pylori* infection and inflammation were retrieved from the GeneCards database (https://www.genecards.org/) by separately searching the keywords “*H. pylori*” and “inflammation.” All retrieved genes were collected, merged, and duplicate entries were removed to obtain the disease-related targets. The overlapping targets between clove-related targets and disease-related targets were visualized using the Venny 2.1.0 online tool.

#### 2.2.2 PPI network construction

The potential antibacterial target genes of clove were imported into the STRING database (https://string-db.org/) to perform protein-protein interaction (PPI) network analysis. The resulting PPI network was downloaded in TSV format and imported into Cytoscape version 3.7.2. Key genes within the network were identified by calculating the degree values.

#### 2.2.3 GO and KEGG enrichment analysis

Gene Ontology (GO) functional enrichment and Kyoto Encyclopedia of Genes and Genomes (KEGG) pathway analyses of the antibacterial targets of clove were performed using the DAVID database (https://davidbioinformatics.nih.gov/).

#### 2.2.4 Molecular docking

Based on the degree values, the top five key targets in the PPI network were selected for molecular docking with the key bioactive components. The 3D structures of the bioactive compounds were retrieved from the PubChem and TCMSP databases, while the crystal structures of the target proteins in PDB format were obtained from the UniProt and Protein Data Bank (PDB; https://www.rcsb.org/). Prior to docking, the bioactive compounds and target proteins were preprocessed using PyMOL version 2.3.0, including hydrogen addition, removal of water molecules, and deletion of charges. Molecular docking was then performed using AutoDock version 4.2.6, and the docking results were subsequently visualized using PyMOL version 2.3.0.

### 2.3 Antimicrobial susceptibility testing

Stock solutions of kaempferol, quercetin, β-sitosterol, stigmasterol, and strictosamide were prepared. Brain Heart Infusion (BHI) defibrinated sheep blood agar medium was prepared by dissolving 26 g of BHI powder and 7.5 g of agar in 500 mL of distilled water, followed by sterilization at 121 °C for 15 min; 5% defibrinated sheep blood was added before use. Drug containing media were prepared by gradient dilution and dispensed into 24 well plates, with the eighth well serving as the control, and nitrocellulose (NC) membranes were placed in each well. After confirmation by 16S rRNA sequencing, *H. pylori* was adjusted to an optical density at 600 nm (OD_600_) of 0.3 and diluted 1,000-fold. An aliquot of 20 μL of the bacterial suspension was spotted onto the NC membranes and incubated at 37 °C under microaerophilic conditions for 7 days. The MIC was defined as the lowest drug concentration at which no visible colony growth was observed on the NC membrane, and bacterial cultures at the MIC and higher concentrations were further assessed to determine the MBC, defined as the lowest concentration yielding fewer than 10 colonies.

### 2.4 Animal model

#### 2.4.1 Grouping and model establishment

Ten rats were randomly selected as the control group, while the remaining 40 rats were used to establish the *H. pylori* infection model. The model was established according to previously reported methods with minor modifications (Tian and Huang, 2019), as follows. A 2.5% amoxicillin solution was administered by oral gavage twice daily at a dose of 0.5 mL per rat for three consecutive days. After a 2-day rest period, rats were gavaged (under fasting conditions) with 0.5 mL per rat of solutions prepared as follows: 0.1 mmol/L NaHCO_3_ solution, 56% ethanol, 5 mg/mL indomethacin solution, and a solution containing 2 g/L NaHCO_3_ plus indomethacin. Food deprivation was maintained after gavage, and 6 h later, *H. pylori* suspension (1.5 mL per rat; bacterial concentration 1 × 10^9^/mL) was administered by gavage. Thereafter, *H. pylori* suspension was administered once every other day for a total of five times. After the final gavage, two rats were randomly sacrificed, and gastric antral mucosa samples were collected for Gram staining and rapid urease testing. Rats showing double positive results were considered successfully colonized with *H. pylori*. Ten rats were randomly assigned to the *H. pylori* infection group, and the remaining rats were divided into low-, medium-, and high-dose clove treatment groups, receiving clove at doses of 0.05, 0.1, and 0.2 g/kg, respectively, by oral gavage once daily (Chinese Pharmacopoeia Commission, 2025; Wang et al., 2023). Rats in the normal control and *H. pylori* infection groups received an equal volume of normal saline by gavage.

#### 2.4.2 Hematological analysis and safety evaluation

Routine blood parameters were measured in rats from each group. Gastric, hepatic, and renal tissues were harvested, weighed, and used to calculate organ coefficients, defined as organ weight divided by body weight. Portions of the tissues were fixed, embedded in paraffin, sectioned, and stained with hematoxylin and eosin (H&E). Slides were sealed and scanned using a panoramic digital slide scanner. Images were captured at 100× and 200× magnifications using CaseViewer version 2.4.0 for histopathological evaluation.

#### 2.4.3 Measurement of inflammatory cytokines in gastric tissue

Approximately 200 mg of gastric tissue from each rat was homogenized in 2 mL of ice-cold normal saline and centrifuged at 3,500 r/min for 5 min. The supernatants were collected, and the concentrations of IL-12, IL-17, IL-23, IFN-γ, and TNF-α were determined using ELISA kits according to the manufacturers’ instructions.

#### 2.4.4 Protein expression analysis in gastric tissue

The expression levels of key target proteins in gastric tissue were analyzed by Western blotting. Gastric mucosal tissues were homogenized in RIPA lysis buffer containing 1 mM PMSF and 1 mM sodium orthovanadate. After centrifugation at 12,000 r/min for 5 min, the supernatants were collected. Protein concentrations were determined using the BCA method. Samples were mixed with 5× loading buffer and boiled for 10 min. Proteins were separated by 12% SDS-PAGE and transferred onto PVDF membranes. Membranes were blocked with TBST containing 5% nonfat milk for 2 h, incubated with primary antibodies at 4 °C overnight, washed with TBST five times (5 min each), and then incubated with secondary antibodies at room temperature for 2 h. Protein bands were visualized using an enhanced chemiluminescence (ECL) system and exposed to X-ray film. GAPDH was used as an internal control, and band intensities were quantified using IPP software.

#### 2.4.5 mRNA expression analysis in gastric tissue

Total RNA was extracted from freshly frozen gastric tissues stored at −80 °C using the TRIzol chloroform method after mechanical homogenization. Complementary DNA (cDNA) was synthesized by reverse transcription. Specific primers were designed for target genes. Quantitative real-time PCR (qPCR) was performed using 5 μL of AceQ qPCR SYBR Green Master Mix with an initial pre-denaturation at 95 °C for 15 min, followed by 40 cycles of amplification at 95 °C for 15 s and 61 °C for 60 s. Melting curve analysis was conducted after amplification to verify product specificity. Each sample was analyzed in triplicate.

### 2.5 Statistical analysis

Statistical analyses were performed using SPSS version 26.0. Normally distributed continuous variables are expressed as the mean ± standard deviation (x̄ ± s), whereas non-normally distributed variables are presented as the median (interquartile range). Comparisons among multiple groups were performed using one-way analysis of variance (ANOVA) for normally distributed data, followed by the least significant difference (LSD) test for *post hoc* pairwise comparisons. For non-normally distributed data, the Kruskal–Wallis H test was used. A p < 0.05 was considered statistically significant.

## 3 Results

### 3.1 Network pharmacology analysis of clove against *Helicobacter pylori*

#### 3.1.1 Identification of potential targets of clove and *Helicobacter pylori*

**FIGURE 1:**
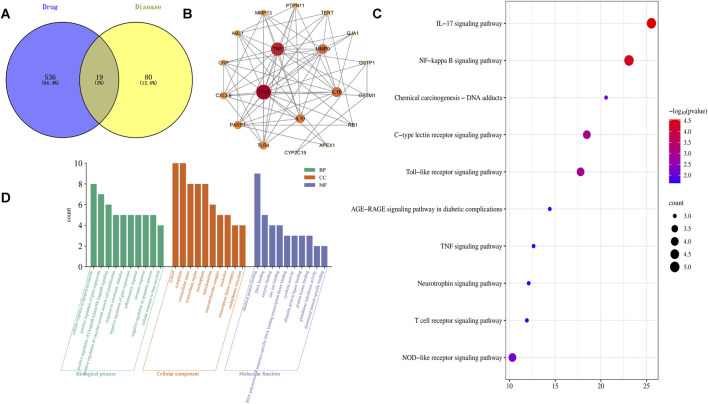
Network pharmacology analysis results. **(A)** Venn diagram of the intersecting genes between clove- and H. pylori–related targets; **(B)** PPI network of the targets involved in the effects of clove against *Helicobacter pylori*; **(C)** GO functional enrichment analysis of key antibacterial targets of clove; **(D)** KEGG pathway enrichment analysis of target genes involved in the inhibitory effects of clove against *Helicobacter pylori*.

A total of six bioactive components were identified in clove, corresponding to 555 potential targets; detailed information is provided in Table 1. Additionally, 99 targets associated with *H. pylori* related disease were collected. Intersection analysis revealed 19 overlapping targets, namely MMP9, MMP13, PARP1, TERT, ABL1, CYP2C19, APEX1, PTPN11, GSTP1, TLR4, CRP, CXCL8, GJA1, GSTM1, IL10, IL1B, RB1, TNF, and TP53, which were considered potential targets through which clove may exert therapeutic effects against *H. pylori* (Figure 1A).

**TABLE 1:** Major bioactive components and targets of clove.

Number	ID	Molecular name	OB ≥30%	DL ≥0.18	No. of targets
1	MOL013219	Strictosamide_qt	76.30	0.76	50
2	MOL000098	Quercetin	46.43	0.28	198
3	MOL000358	Beta-sitosterol	36.91	0.75	68
4	MOL000422	Kaempferol	41.88	0.24	100
5	MOL000449	Stigmasterol	43.83	0.76	41
6	MOL001749	ZINC03860434	43.59	0.35	98

#### 3.1.2 Construction of the PPI network

A PPI network of the 19 overlapping targets was constructed using the STRING database (Figure 1B). The network consists of 19 nodes and 62 interaction edges, with an average node degree of 7.58. A higher number of connections corresponds to a greater degree value, indicating the increased importance of the target within the network. The top five targets ranked by degree were TP53, MMP9, IL1B, IL10, and TNF. Notably, TP53 exhibited a degree value more than twice the network average.

#### 3.1.3 Results of GO and KEGG enrichment analyses

GO and KEGG enrichment analyses were performed for the five key targets. A total of 152 biological process (BP), 16 cellular component (CC), and 18 molecular function (MF) terms were identified (Figure 1C), along with 64 enriched signaling pathways (Figure 1D). The BP terms were mainly associated with positive regulation of vascular smooth muscle cell proliferation, inflammatory responses, and immune responses. The CC terms primarily involved the extracellular space, extracellular region, and macromolecular complexes. The MF terms included RNA polymerase II sequence-specific DNA-binding transcription factor binding and cytokine activity. Enriched pathways included the IL-17 signaling pathway, NF-κB signaling pathway, C-type lectin receptor signaling pathway, Toll-like receptor signaling pathway, NOD-like receptor signaling pathway, chemical carcinogenesis–DNA adducts, and the tumor necrosis factor signaling pathway. These pathways mainly involved targets such as IL10, IL1B, MMP9, TNF, TP53, and TLR4.

#### 3.1.4 Results of molecular docking analysis

**FIGURE 2:**
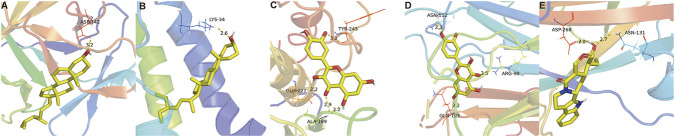
Optimal molecular docking models between key antibacterial targets and bioactive components of clove. **(A)** β-sitosterol with IL1B; **(B)** stigmasterol with IL10; **(C)** quercetin with MMP9; **(D)** quercetin with TNF; **(E)** strictosamide with TP53.

The major bioactive components of clove were docked with the top five key targets in the PPI network ranked by degree value. The docking results showed that all compound target complexes exhibited minimum binding energies below zero, indicating favorable binding interactions (Table 2). Among these, quercetin displayed the lowest binding energy with MMP9. The bioactive compound with the optimal docking score for each target protein was selected for visualization (Figure 2). Distinct binding sites were observed between different target proteins and bioactive components. Specifically, quercetin formed hydrogen bond interactions with MMP9 at the ALA-189, GLU-227, and TYR-245 residues, whereas its interaction with TNF involved hydrogen bonding at the GLN-102, ARG-98, and ASN-112 residues.

**TABLE 2:** Molecular docking results of key antibacterial targets and bioactive components of clove.

PDB ID	Core target	Binding energy (kcal/mol)					
		Quercetin	Beta-sitosterol	Kaempferol	Stigmasterol	ZINC03860434	Strictosamide_qt
2E7A	TNF	−7.8	−8.1	−9.0	−7.4	−6.7	−7.4
1INR	IL10	−6.5	−8.3	−6.4	−9.0	−6.0	−7.8
4AGP	TP53	−6.4	−6.7	−6.5	−6.8	−5.0	−7.2
6I8Y	IL1B	−6.3	−7.2	−6.3	−7.0	−5.4	−6.6
4XCT	MMP9	−10.5	−8.6	−9.9	−9.1	−7.1	−8.8

### 3.2 *In vitro* antibacterial activity of clove against *Helicobacter pylori*

Based on visual assessment of bacterial growth, the MIC of quercetin, kaempferol, and stigmasterol was 2.5, 0.5, and 3.13 mg/mL, respectively, while their corresponding MBC were 10, 1, and 12.52 mg/mL. The MIC of strictosamide was 10 mg/mL; however, its MBC was not detected under the experimental conditions. In contrast, β-sitosterol did not exhibit quantifiable MIC or MBC values within the concentration range tested in this study.

### 3.3 *In vivo* anti-inflammatory effects and mechanisms of clove against *Helicobacter pylori*

#### 3.3.1 Hematological parameters and organ safety in rats

##### 3.3.1.1 Routine blood analysis

Compared with the control (CON) group, the *H. pylori* group showed significant increases in several hematological parameters, including white blood cell count, neutrophil count, lymphocyte count, and monocyte count. Compared with the *H. pylori* group, clove treatment significantly reduced some inflammatory hematological parameters, particularly neutrophil and monocyte counts, whereas changes in other indices varied among dose groups. Platelet-related indices such as PDW also differed significantly among groups. The detailed results are presented in Table 3.

**TABLE 3:** Hematological parameters in different rat groups.

Parameter	CON	Hp	High dose	Medium dose	Low dose	F/H value	p value
WBC	0.677 ± 0.247	3.227 ± 0.186^#^	1.185 ± 0.341^#^	1.625 ± 0.309^#*a^	2.768 ± 0.365^*a^	40.122^1^	0
NEU	0.080 ± 0.036	0.590 ± 0.213^#^	0.203 ± 0.135^*^	0.190 ± 0.064^*^	0.275 ± 0.086^*^	8.155^1^	0.002
LYM	0.513 ± 0.155	1.763 ± 0.428^#^	0.748 ± 0.228^#^	1.198 ± 0.216^#*a^	2.095 ± 0.328^*a△^	19.754^1^	0
MONO	0.057 ± 0.074	0.823 ± 0.146^#^	0.210 ± 0.065^#*^	0.203 ± 0.094^*^	0.3050 ± 0.060^#*^	33.634^1^	0
EOS	0.020 (0.010,0.050)	0.060 (0.030,0.060)	0.075 (0.045,0.098)^#^	0.010 (0.010,0.025)^*^	0.010 (0.010,0.040)	10.929^2^	0.027
BASO	-	-	0.015 (0.003,0.043)	0.020 (0.003,0.037)	0.000 (0.000,0.015)	7.686^2^	0.104
NEU%	10.900 ± 3.7242	18.133 ± 6.850	16.125 ± 7.557	11.550 ± 2.034	10.000 ± 2.787	1.730^1^	0.203
LYM%	76.333 ± 4.796	54.467 ± 12.217^#^	62.725 ± 11.581^*^	73.700 ± 5.046^*^	75.500 ± 4.944^a^	4.385^1^	0.018
MONO%	7.233 ± 6.6161	25.633 ± 5.5320^#^	18.150 ± 5.314^*^	12.175 ± 4.713^*^	10.975 ± 1.900^#^	6.840^1^	0.003
EOS%	2.200 (2.100,9.900)	1.900 (1.100,2.000)	2.500 (1.825,4.150)	0.850 (0.725,1.575)	2.000 (1.375,3.600)	9.070^2^	0.059
BASO%	0.200 (0.200,2.000)	0.100 (0.100,0.100)	0.650 (0.100,1.350)	1.300 (0.275,3.000)	0.450 (0.175,1.400)	4.720^2^	0.317
RBC	4.447 ± 3.612	6.89 ± 0.617	6.108 ± 1.413	6.730 ± 0.852	3.943 ± 2.280	1.677^1^	0.215
Hb	118.00 ± 30.199	144.330 ± 13.868	126.25 ± 29.971	150.50 ± 6.856	130.50 ± 5.066	1.565^1^	0.242
HCT	28.567 ± 23.312	45.033 ± 5.064	40.600 ± 9.911	44.575 ± 7.548	26.050 ± 15.302	1.646^1^	0.222
MCV	63.700 (57.000,65.400)	64.900 (62.900,68.000)	65.700 (63.350,69.025)	67.650 (60.775,69.725)	66.850 (65.000,67.050)	2.716^2^	0.606
MCH	21.800 (19.900,215.400)	20.900 (20.800,21.100)	36.350 (21.625,82.875)	22.050 (20.375,25.525)	20.850 (19.925,21.250)	6.014^2^	0.198
MCHC	333.000 (313.000,3777.000)	320.000 (311.000,333.000)	566.500 (325.250,1234.500)	322.500 (299.000,418.750)	311.500 (306.250,318.250)	6.914^2^	0.141
RDW-CV	27.300 ± 11.820	17.967 ± 0.896	17.600 ± 1.273	25.825 ± 10.875	26.500 ± 9.578	1.167^1^	0.37
RDW-SD	65.800 ± 22.842	47.833 ± 4.5369	47.650 ± 4.425	66.950 ± 22.907	69.400 ± 23.231	1.275^1^	0.329
PLT	394.00 ± 89.404	784.33 ± 256.752^#^	715.250 ± 227.917	474.50 ± 179.102	714.250 ± 254.740	2.136^1^	0.134
MPV	8.633 ± 1.401	8.033 ± 0.666	7.675 ± 0.263	7.375 ± 1.124	8.150 ± 0.998	0.882^1^	0.501
PDW	18.200 ± 1.4107	16.600 ± 0.592^#^	17.250 ± 0.661^*^	16.550 ± 0.379^#a^	17.900 ± 0.627	3.310^1^	0.045
PCT	0.348 ± 0.137	0.633 ± 0.234	0.544 ± 0.162	0.346 ± 0.137	0.589 ± 0.238	1.834^1^	0.183

^#^indicates p < 0.05 vs. control group; *indicates p < 0.05 vs. *H. pylori* infected group; ^a^indicates p < 0.05 vs. high-dose group; ^△^indicates p < 0.05 vs. medium-dose group; ^1^denotes one-way ANOVA (F test), and ^2^denotes nonparametric test (H test). WBC, white blood cell count; NEU, neutrophil count; LYM, lymphocyte count; MONO, monocyte count; EOS, eosinophil count; BASO, basophil count; RBC, red blood cell count; Hb, Hemoglobin; HCT, hematocrit; MCV, mean corpuscular volume; MCH, mean corpuscular hemoglobin; MCHC, mean corpuscular hemoglobin concentration; RDW-CV, Red Blood Cell Distribution Width–Coefficient of Variation; RDW-SD, Red Blood Cell Distribution Width–Standard Deviation; PLT, platelet count; MPV, mean platelet volume; PDW, platelet distribution width; PCT, plateletcrit.

##### 3.3.1.2 Gastric, hepatic, and renal organ indices

Significant intergroup differences were observed in the gastric, hepatic, and renal organ indices of rats across the experimental groups. With respect to the hepatic index, the *H. pylori* infection group showed a significant decrease compared with the control group. The medium dose group exhibited a hepatic index that was lower than that of the control group but significantly higher than that of the *H. pylori* infection group. Regarding the renal index, the *H. pylori* infection group as well as the low and high dose groups showed significantly lower values than the control group. In terms of the gastric index, the high dose group demonstrated a significantly higher value than both the *H. pylori* infection group and the medium dose group. No statistically significant differences in organ indices were observed among the remaining group comparisons. The results are presented in Table 4.

**TABLE 4:** Organ indices of gastric, hepatic, and renal tissues in different rat groups.

Organ index	CON	Hp	Low dose	Medium dose	High dose
Liver	3.12 ± 0.30	2.46 ± 0.16*	2.67 ± 0.37	2.69 ± 0.10^*△^	2.51 ± 0.12^*^
Kidney	0.71 ± 0.08	0.60 ± 0.07*	0.59 ± 0.06*	0.62 ± 0.06	0.59 ± 0.06*
Stomach	0.57 ± 0.05	0.52 ± 0.05	0.59 ± 0.03	0.52 ± 0.04	0.61 ± 0.04^#△^

*indicates p < 0.05 vs. control group; ^△^indicates p < 0.05 vs. Hp group; ^#^indicates p < 0.05 vs. medium-dose group.

##### 3.3.1.3 Histopathological findings of gastric, hepatic, and renal tissues

**FIGURE 3:**
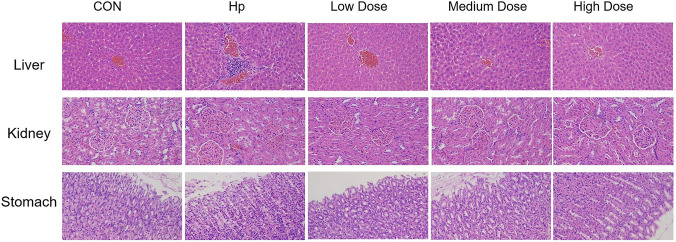
Histopathological observations of gastric, hepatic, and renal tissues by H&E staining.

In the control group, the gastric, hepatic, and renal tissues exhibited normal histological architecture. In the *H. pylori* infection group, the gastric tissue showed mild cellular disorganization and focal cell detachment, occasional inflammatory cell aggregation was observed in the liver, whereas no obvious abnormalities were detected in the kidney. All clove treated groups exhibited varying degrees of tissue repair. In gastric tissues, only sporadic mild cell detachment was observed, with no evident inflammatory infiltration or mucosal damage. In the high dose group, both hepatic and renal tissues returned to normal histological morphology. The medium dose group showed no apparent abnormalities in liver or kidney structure. In the low dose group, hepatic and renal tissues also appeared morphologically normal, and gastric tissue repair was stable, with overall inflammatory damage markedly improved compared with the *H. pylori* infection group (shown in Figure 3).

### 3.4 Effects of clove on gastric inflammation

ELISA results showed that the concentrations of proinflammatory cytokines, including TNF-α, IL-12, IL-17, IL-23, and IFN-γ, were significantly increased in the gastric tissues of *H. pylori* infected rats. Following clove intervention, the levels of these proinflammatory cytokines were markedly reduced, exhibiting a clear dose dependent pattern. The high-dose clove group showed the most pronounced reductions, followed by the medium dose group, whereas the low dose group demonstrated comparatively weaker effects. The results are summarized in Table 5.

**TABLE 5:** Inflammatory cytokine levels in gastric tissues of different rat groups (pg/mL).

Group	n	TNF-α	IL-12	IL-17	IL-23	IFN-γ
Control	10	167.252 ± 21.541	250.844 ± 24.025	310.501 ± 40.126	266.334 ± 31.285	330.082 ± 35.521
Hp	10	741.432 ± 79.173^#^	1265.644 ± 112.534^#^	1576.436 ± 176.963^#^	1309.677 ± 133.504^#^	1647.395 ± 153.591^#^
High dose	10	289.526 ± 41.062^*^	497.785 ± 52.299^#*^	601.342 ± 83.527^#*^	544.040 ± 67.040^#*^	563.325 ± 71.418^#*^
Medium dose	10	458.605 ± 53.377^#*a^	754.206 ± 73.092^#*a^	937.632 ± 117.625^#*a^	824.613 ± 89.599^#*a^	998.607 ± 100.810^#*a^
Low dose	10	586.365 ± 57.205^#*a△^	1023.167 ± 83.585^#*a△^	1276.371 ± 116.998^#*a△^	1140.767 ± 96.088^#*a△^	1406.587 ± 162.151^#*a△^

^#^indicates p < 0.05 vs. control group; ^*^ indicates p < 0.05 vs. Hp group; ^a^ indicates p < 0.05 vs. high-dose group; ^△^ indicates p < 0.05 vs. medium-dose group.

### 3.5 Effects of clove on inflammation related molecules in gastric tissue

**FIGURE 4:**
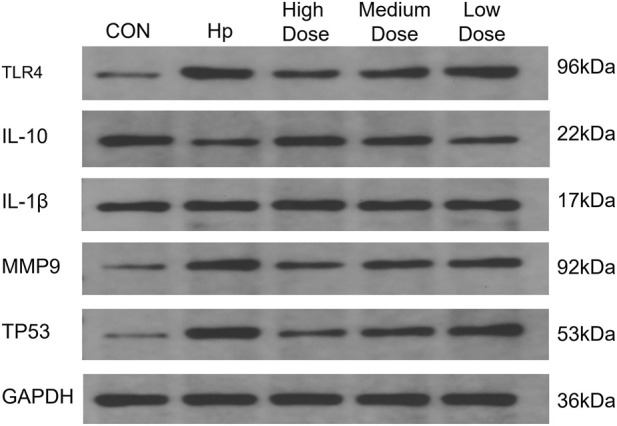
Representative protein expression bands of IL-10, IL-1β, MMP9, TP53, and TLR4 in gastric tissues of rats from different groups.

Western blot analysis revealed that compared with the normal control group, gastric tissues from the *H. pylori* infected group exhibited significantly decreased IL-10 protein levels along with markedly elevated expression of IL-1β, MMP9, TP53, and TLR4, indicating that *H. pylori* infection successfully induced pronounced inflammatory responses and tissue injury. After clove intervention, the high dose group showed a significant increase in IL-10 expression and a clear pronounced suppression of IL-1β, MMP9, TP53, and TLR4 levels compared with the *H. pylori* infection group. The effects in the medium-dose group were less pronounced than those in the high-dose group, although IL-10 expression was still significantly increased compared with the *H. pylori* group. Notably, TLR4 expression was significantly suppressed even in the low-dose group and was lower than that in the medium dose group. These results are summarized in Table 6 and Figure 4.

**TABLE 6:** Protein expression levels in gastric tissues of different rat groups.

Group	n	IL-10	IL-1β	MMP9	TP53	TLR4
Control	10	0.813 ± 0.0913	0.131 ± 0.029	0.179 ± 0.046	0.223 ± 0.069	0.198 ± 0.047
Hp	10	0.327 ± 0.052^#^	0.596 ± 0.067^#^	0.772 ± 0.075^#^	0.919 ± 0.055^#^	0.866 ± 0.129^#^
High dose	10	0.705 ± 0.091^*^	0.302 ± 0.044^#*^	0.357 ± 0.062^#*^	0.413 ± 0.099^#*^	0.722 ± 0.047^#*^
Medium dose	10	0.524 ± 0.103^#*a^	0.404 ± 0.016^#*a^	0.529 ± 0.030^#*a^	0.586 ± 0.0529^#*a^	0.553 ± 0.074^#*a^
Low dose	10	0.356 ± 0.058^#a△^	0.559 ± 0.044^#a△^	0.646 ± 0.076^#*a△^	0.775 ± 0.028^#*a△^	0.383 ± 0.038^#*a△^

^#^indicates p < 0.05 vs. control group; ^*^indicates p < 0.05 vs. Hp group; ^a^indicates p < 0.05 vs. high-dose group; ^△^indicates p < 0.05 vs. medium-dose group.

Consistent with the protein expression finding, quantitative real-time PCR analysis further demonstrated a significant downregulation of IL-10 mRNA expression in the gastric tissues of the *H. pylori* infection group compared with the normal control group, accompanied by marked upregulation of IL-1β, MMP9, TP53, and TLR4 mRNA levels. Following clove treatment, the high-dose group exhibited a pronounced restoration of IL-10 expression along with the strongest inhibitory effects on IL-1β, MMP9, TP53, and TLR4 expression. The medium-dose group also showed significant improvements in these parameters; however, the expression levels of IL-1β, MMP9, TP53, and TLR4 remained significantly higher than those in the high-dose group. Although the low-dose group significantly suppressed IL-1β and TP53 expression, it did not substantially restore IL-10 expression, and its inhibitory effects on MMP9 and TLR4 were weaker than those observed in the medium- and high-dose groups. The results are presented in Table 7.

**TABLE 7:** mRNA expression levels in gastric tissues of different rat groups.

Group	n	IL-10	IL-1β	MMP9	TP53	TLR4
Control	10	1.083 ± 0.107	1.014 ± 0.186	1.023 ± 0.193	1.025 ± 0.177	1.014 ± 0.19
Hp	10	0.215 ± 0.077^#^	5.341 ± 0.452^#^	4.966 ± 0.463^#^	4.707 ± 0.472^#^	5.181 ± 0.24^#^
High dose	10	0.578 ± 0.120^#*^	4.528 ± 0.397^#*^	2.050 ± 0.426^#*^	2.301 ± 0.332^#*^	1.853 ± 0.386^#*^
Medium dose	10	0.486 ± 0.073^#*a^	3.277 ± 0.459^#*a^	2.931 ± 0.419^#*a^	3.096 ± 0.340^#*a^	3.134 ± 0.325^#*a^
Low dose	10	0.335 ± 0.100^#a△^	1.935 ± 0.377^#*a△^	4.188 ± 0.424^#*a△^	3.808 ± 0.495^#*a△^	4.21 ± 0.298^#*a△^

^#^indicates p < 0.05 vs. control group; ^*^indicates p < 0.05 vs. Hp group; ^a^indicates p < 0.05 vs. high-dose group; ^△^indicates p < 0.05 vs. medium-dose group.

## 4 Discussion

As a pathogenic bacterium closely associated with digestive diseases, *H. pylori* poses substantial challenges to existing therapeutic strategies because of its high global prevalence and the growing problem of antibiotic resistance. Therefore, identifying novel, effective, and safe therapeutic agents has become an important research priority. Clove, a commonly used Chinese and Mongolian medicinal herb, has long been applied in traditional medicine for the management of digestive disorders (Maggini et al., 2024). Modern studies have shown that its constituents can alleviate tissue inflammatory injury by regulating inflammatory mediators and exhibit *in vitro* inhibitory activity against various pathogenic bacteria, including *Staphylococcus aureus* and *Escherichia coli* (Peng et al., 2023; Kaur et al., 2024). However, current research on the anti-*H. pylori* effects of clove remains limited, and its systematic mechanisms of action have not been fully elucidated. Therefore, the present study established an integrated framework combining target prediction, molecular docking, *in vitro* antimicrobial susceptibility testing, and *in vivo* pharmacodynamic evaluation to investigate the anti-*H. pylori* related effects of clove.

To preliminarily explore the potential mechanism of clove against *H. pylori*, network pharmacology analysis identified six core bioactive components and 19 key targets associated with *H. pylori* infection. Among these, TP53, MMP9, IL1B, IL10, and TNF emerged as central nodes in the network. Functional enrichment analysis indicated that these targets were mainly involved in biological processes related to inflammatory responses and immune regulation, with significant enrichment in pathways such as the TNF signaling pathway and the Toll-like receptor signaling pathway. These findings suggest that the anti-*H. pylori* effects of clove may be associated with modulation of inflammation-related pathways. Molecular docking further suggested that key components, such as quercetin and kaempferol, may interact with these core targets, providing preliminary, hypothesis-generating evidence for the potential antibacterial and anti-inflammatory activities of clove in the context of *H. pylori* infection. Based on these findings, *in vitro* antimicrobial susceptibility assays were conducted to verify the direct antibacterial activity of clove-derived bioactive components. The results showed that quercetin, kaempferol, and stigmasterol exhibited clear inhibitory effects against the *H. pylori* SS1 strain, with kaempferol showing the strongest antibacterial activity. In contrast, strictosamide exhibited only weak antibacterial activity, while β-sitosterol did not yield quantifiable inhibitory concentrations within the tested range. These findings not only help explain inconsistencies in previously reported antibacterial concentrations of clove constituents arising from differences in extraction procedures and detection methods but also provide evidence supporting the anti-*H. pylori* activity of stigmasterol, thereby expanding the repertoire of active anti-*H. pylori* constituents in clove. Collectively, these *in vitro* results support direct anti-*H. pylori* activity of several clove-derived components under the present experimental conditions. This is broadly consistent with recent studies on plant-based anti-*H. pylori* interventions, which suggest that natural products may exert protective effects through combined antibacterial, anti-inflammatory, and mucosal protective actions rather than through a single mechanism alone (Wu et al., 2025). Peng et al. (2024) reported that *S. aromaticum* aqueous extract inhibited *H. pylori* and was associated with gastric mucosal protection through multi-target regulation, whereas Elbestawy et al. (2023) demonstrated that clove-derived essential oil exhibited antibacterial, antibiofilm, and anti-inflammatory activities against resistant *H. pylori*. Compared with these previous studies, the present work further identifies specific clove-derived constituents, including kaempferol, quercetin, and stigmasterol, as candidate compounds associated with direct *in vitro* anti-*H. pylori* activity. However, whether these effects translate into *in vivo* antibacterial activity remains to be determined.

Animal experiments showed that *H. pylori* infection induced multiple pathological abnormalities, particularly significant elevations in proinflammatory cytokines, including TNF-α, IL-12, IL-17, IL-23, and IFN-γ, together with downregulation of the anti-inflammatory cytokine IL-10. This overall pattern is also consistent with the broader pharmacological profile of clove summarized in recent reviews, which describe clove and its major constituents as possessing both antimicrobial and anti-inflammatory properties (Pandey et al., 2024). In the present study, this imbalance was significantly reversed by clove treatment in a dose-dependent manner. Further analyses suggested that clove exerted coordinated anti-inflammatory and tissue-protective effects, with possible contributions from its antibacterial activity observed *in vitro*. Western blotting and qPCR analyses showed that clove significantly reduced the aberrantly elevated protein and mRNA expression levels of IL-1β, MMP9, TP53, and TLR4 in H. pylori-infected models. These factors are closely involved in inflammation, tissue injury, and host pathogen responses during *H. pylori* infection.

Clove intervention significantly reduced IL-1β expression in the high-dose group, although the level remained higher than that in the control group. IL-1β, a key inflammatory mediator in *H. pylori* infection, has been shown to damage the gastric mucosa and promote inflammatory infiltration upon release (Tran et al., 2023). A clinical study by Zhao et al. (2013) also reported significantly higher serum IL-1β levels in *H. pylori* positive patients than in *H. pylori* negative individuals, with levels positively correlated with the severity of gastritis. These findings suggest that reduction of IL-1β expression may contribute to the protective effects of clove and support IL-1β as a plausible inflammation-related target associated with its action. Analysis of MMP9 expression further showed that clove inhibited MMP9 in a dose-dependent manner, suggesting that MMP9 may be an important mediator of *H. pylori* induced tissue damage. Overexpression of MMP9 can degrade the extracellular matrix and disrupt the gastric mucosal barrier (Sokolova and Naumann, 2022). Although Peng et al. (2024) previously hypothesized that clove might protect the gastric mucosa by regulating such factors, direct target-based evidence was lacking. The present results suggest that suppression of MMP9 may contribute, at least in part, to preservation of mucosal barrier integrity. This effect may complement the *in vitro* antibacterial activity of specific clove-derived compounds in limiting *H. pylori* related injury. With respect to TP53 regulation, this study further showed that clove dose-dependently suppressed its aberrant overexpression. TP53, a critical tumor suppressor gene (Zhou et al., 2025), is closely associated with the progression of *H. pylori*-related pathological changes when dysregulated. Matsumoto et al. (2007) reported that *H. pylori* infection could promote the accumulation of mutations in the TP53 tumor suppressor gene through aberrant AID expression, thereby contributing to gastric carcinogenesis. In the present study, clove reduced *H. pylori* associated TP53 overexpression, suggesting that it may influence genes related to TP53 signaling in the infected gastric mucosa. This observation broadens the possible mechanistic relevance of clove, although its biological significance requires further study in the context of long-term disease progression.

Furthermore, KEGG enrichment analysis identified the TLR4 signaling pathway as a key pathway enriched among the core targets of clove. Experimental validation confirmed that TLR4 expression was significantly upregulated in the *H. pylori* infection model, whereas clove intervention suppressed its expression in a dose-dependent manner. As a key pattern recognition receptor for *H. pylori*, TLR4 activation initiates the NF-κB signaling pathway and promotes the release of proinflammatory cytokines (Gao et al., 2025). Previous studies have demonstrated that excessive activation of this pathway is a critical driver of progression from *H. pylori* associated gastritis to gastric cancer (Meliț et al., 2019; Zhang Z. et al., 2025). The present findings indicate that clove was associated with reduced TLR4 expression in the *H. pylori* infection model together with decreased downstream proinflammatory cytokine levels. These results suggest that clove may modulate inflammation-related molecules associated with the TLR4/NF-κB axis. However, because NF-κB activation, nuclear translocation, and phosphorylation status were not directly assessed in the present study, the data should be interpreted as supporting potential involvement of this pathway rather than definitive pathway regulation.

Nevertheless, several limitations should be acknowledged. First, the network pharmacology and molecular docking analyses were exploratory and database dependent and therefore provide hypothesis generating rather than definitive mechanistic evidence. Second, direct quantification of bacterial colonization or bacterial load in gastric tissue was not performed, and no conventional positive control group was included in the animal experiment; therefore, the *in vivo* findings more directly support anti-inflammatory and gastroprotective effects than a definitive *in vivo* antibacterial mechanism and cannot be directly compared with established anti-*H. pylori* therapies. Third, the *in vitro* antibacterial assays were conducted using a single *H. pylori* strain (SS1), which may limit generalizability. Fourth, isolated compounds were evaluated *in vitro*, whereas whole clove was administered *in vivo*, and the clove preparation used *in vivo* was not chemically characterized in detail, leaving the link between compound level activity and whole herb effects indirect. Future studies should address these issues through phytochemical characterization, broader strain validation, direct *in vivo* bacterial quantification, inclusion of appropriate positive controls, and functional pathway assays.

In summary, the present findings suggest that clove exerts anti-*H. pylori* related effects through direct *in vitro* antibacterial activity together with *in vivo* anti-inflammatory and gastroprotective actions. These effects may involve TNF- and TLR4-associated signaling, although further studies are needed to verify direct pathway regulation and *in vivo* antibacterial mechanisms.

## Data Availability

The data presented in this study are available on reasonable request from the corresponding author due to privacy and ethical restrictions.
